# Dementia Caregivers Perspectives of Caregiving, Burden, Emergencies, and Emergency Preparedness

**DOI:** 10.1093/geroni/igaa057.3337

**Published:** 2020-12-16

**Authors:** Nicole Crawford, Bruce Levin, Gum Amber, Dinorah Martinez-Tyson, Makeba Huntington-Symons, Anthony Masys

**Affiliations:** 1 University of South Florida, Tampa, Florida, United States; 2 Alzheimer’s Association, Clearwater, Florida, United States

## Abstract

Limited research has been conducted regarding the perspectives of caregivers of persons with dementia (PWD) regarding preparing for emergencies. The purpose of this study was to understand dementia caregivers’ perceptions of burden, emergencies, and emergency preparedness through the caregiving lens. Fourteen semi-structured interviews were conducted with a purposive sample of dementia caregivers who reside in Hillsborough County, Florida, to gain participants’ perspectives. Data were analyzed using the thematic analysis method. Caregivers discussed emergencies in terms of medical issues, mostly related to the PWD’s health, as well as natural disasters such as hurricanes. Caregivers discussed their perspectives of caregiving and burden as centered around their roles and experiences with the PWD, including ways in which they would deal with emergencies such as natural disasters. Caregivers reported that they were physically prepared for emergencies (i.e., hurricanes) but not mentally prepared for coping with or helping their loved ones cope with the stress of an emergency related event. The interviews presented opportunities to raise awareness of emergency preparedness resources and provide information specific to each caregivers’ situation. The results suggest the caregiver role may be essential to mitigating the adverse effects of emergencies on PWDs. Recommendations for practice include providing person-centered care, individualized emergency planning, and interprofessional collaboration.

